# Auditory assessment of alcoholics in abstinence

**DOI:** 10.1016/S1808-8694(15)30097-5

**Published:** 2015-10-19

**Authors:** Sandra Beatriz Afonso Ribeiro, Lilian Cassia Bornia Jacob, Kátia de Freitas Alvarenga, Jair Mendes Marques, Rafaela Mocellin Campêlo, Samira Natacha Tschoeke

**Affiliations:** a M.S. in Communications disorders - Universidade Tuiuti do Paraná.; b PhD in Human communications disorders - USP, Professor of Speech and Hearing Therapy and of the Postgraduate Program on Human Communications Disorders - Universidade Tuiuti do Paraná.; c Professor of Speech and Hearing Therapy – School of Dentistry - Bauru – University of São Paulo (FOB_USP_BAURU).; d Professor of Speech and Hearing Therapy and of the Postgraduate Program on Human Communications Disorders - Paraná Tuiuti University.; e M.S. in Communications disorders - Paraná Tuiuti University.; f M.S. Student in Communications disorders - Paraná Tuiuti University. Universidade Tuiuti do Paraná.

**Keywords:** alcoholism, hearing, diagnosis, hearing loss

## Abstract

Alcoholism is considered the most relevant addiction in the international arena and few investigations have examined the association between sensorineural hearing loss and alcohol abuse, with conflicting results.

**Aim:**

To analyze the effects of alcohol abuse on the auditory system of alcoholics in abstinence taking into account the duration of alcohol abuse and associated noise exposure.

**Method:**

our series comprehended 75 individuals, divided into two groups: trial and control. The audiological assessment was made by means of: pure-tone audiometry, transient evoked otoacoustic emissions, tympanometry. The Wilcoxon and Mann-Whitney tests were used in the statistical analysis of the data.

**Results:**

the groups of patients who had been alcoholics evidenced a statistically significant worse performance in the audiological assessment. The combined exposure to alcohol and noise was not synergic on the auditory system.

**Conclusion:**

long-term alcohol abuse can damage the cochlear function, specifically the outer hair cells.

## INTRODUCTION

Alcoholism or alcohol abuse is considered as a serious issue in Latin America, in the United States of America and in some parts of Europe.1 The World Health Organization has debated alcohol abuse since the 1950s, and alcoholism was included in the International Classification of Diseases in 1967 (ICD-8) during the 8th World Conference on Health.^2^

Lukomski et al.^3^ have noted an increase in drug abuse worldwide, including alcohol. Based on this perspective, other studies have divulged the harmful effects of alcohol on the body when used frequently and at high concentrations; these studies have also shown that alcoholism may be found at any age.^4^, ^5^

The specialized literature in this area presents conflicting results about the effects of chronic alcoholism on hearing; there is no consensus about the toxic potential of alcohol on the auditory system. Furthermore, an overview of the literature shows that there are few published papers on this theme in Brazil.

Alcoholism-related sensorineural hearing loss has been documented in many papers;^1^, ^3^, ^5^, ^6^, ^7^, ^8^, ^9^, ^10^, ^11^, ^12^, ^13^, ^14^, ^15^, ^16^, ^17^, ^18^, ^19^, ^20^, ^21^, ^22^ the methods, however, raise concerns about whether variables such as age, duration of alcohol abuse, and past and present noise exposure might also have contributed to the observed hearing loss.

Brajevic et al.,^23^ and Alpert and Bogorad,^24^ on the other hand, reported a significant correlation between the duration of alcohol abuse and hearing loss in their sample populations. No relation between hearing loss and alcohol abuse was reported only in studies by Nordahl^25^ and Jones et al.^26^ Nordahl^25^ attributed hearing loss to noise exposure.

Tinnitus has also been reported as a symptom in this condition; Spitzer and Ventry,^13^ Spitzer,^15^ Gross et al.^27^ and Quick^28^ thus confirmed alcohol as being toxic to the auditory system.

A review of the Brazilian and international literature on the relation between alcohol abuse and auditory findings revealed a paucity of studies with control and study groups; the present paper is a case control study. Niedzielska et al.^5^ investigated 30 alcoholics by using transient evoked otoacoustic emissions (TEOAE) and noted an absence of TEOAE in 77% of those subjects; they attributed these results to the harmful effects of alcohol on outer hair cell function.

The aim of this paper was to analyze the effect of alcohol on the auditory system of abstemious alcoholics, looking at the variables time of use of alcohol and noise exposure.

## MATERIAL AND METHOD

The Research Ethics Committee approved the study design (Of. CEP nº 102/2003). All of the subjects were informed about the study procedures and signed a free informed consent form.

The design was a cross-sectional case controlled study of the incidence done with a sample of 75 subjects paired into two study groups (GE1 and GE2) and two control groups (GC1 and GC2). The subdivision into four groups became necessary, given a high rate of alcoholics involved in professional activities with significant noise exposure in the working environment.

Subjects diagnosed as alcoholics were referred by their medical doctors to the Psychosocial Support Center for Alcoholics (Centro de Apoio Psicossocial ao Alcoolatra), which is headquartered in Curitiba, PR. The multidisciplinary team confirms the diagnosis based on International Classification of Diseases (ICD-10) criteria.

Inclusion criteria for the GE1 were:

1. subjects diagnosed as alcoholics that drank more than one liter of alcoholic beverages per day;
2. subjects in a period of alcohol abstinence;
3. absence of external or middle ear conditions;
4. a negative history of past or current exposure to noise or chemical products;
5. subjects with no drug dependence other than alcohol
6. normal otoscopy and bilateral type A tympanogram.

GE1 contained 18 subjects, 12 male and six female, aged between 34 and 60 years (mean 46.3 years).

Inclusion criteria for GE2 were the same as those for GE1 except for a history of noise exposure. GE2 contained 22 subjects, 21 male and one female, aged between 29 and 59 years (mean 45.7 years).

Inclusion criteria form GC1 were the same as those for GE1 except for alcohol intake. GC1 contained 12 subjects, six male and six female, aged between 35 and 57 years (mean 45.4 years).

GC2 contained 23 subjects, 19 male and four female, aged between 29 and 67 years (mean 45.5 years) based on the same inclusion criteria as those for GC1 except for a history of noise exposure.

All of the groups were matched for age; groups that were exposed to noise (GE2 and GC2) were also matched for the duration of noise exposure and profession, all of which had similar sound pressure levels (85 to 92dBNPS).

The level of noise in the working environment was obtained by studying the company documents for each worker.

**Chart 1:**
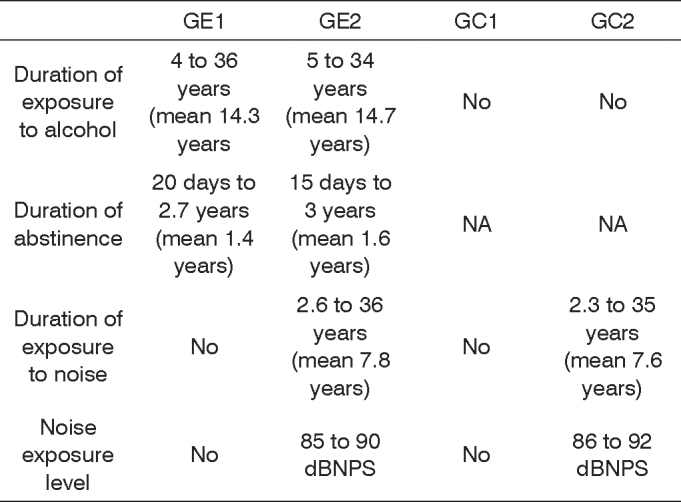
Distribution of time of alcohol abuse, duration of abstinence from alcohol and noise exposure in the study and control groups.

Information about the duration of alcohol abuse, the period of abstinence and the duration of noise exposure in the study groups (GE1 and GE2) and the control groups (GC1 are GC2) presented on Chart 1.

**Key:** GE1 - study group 1; GE2 - study group 2; GC1 - control group 1; GC2 - control group 2; NA - not applicable.

Subjects that used medication (antidepressive and psychotropic medication or ototoxic drugs), that had systemic diseases or a history of otological disease, were excluded from the study.

### Testing Method

Audiological testing was done as follows:

- Pure tone audiometry (PTA)

PTA was done by air conduction at 0.25 to 8kHz and by bone conduction at 0.5 to 4kHz, and the speech recognition threshold. Testing was carried out in an acoustic booth using a model AC40 Interacoustics audiometer, TDH-39 headphones and an MX-41 pad, calibrated according to the ISO 8253/ IEC 645/ ISSO 389 standards.

- Transient Evoked Otoacoustic Emissions Testing (TEOAE)

TEOAE testing was done using an Otodynamics Ltda ILO96 Research OAE System device. Testing was carried out at 1 to 5kHz, although analysis was made based on recordings at 1 to 4kHz, as in practice TEOAE are frequently absent at 5kHz in adults and elderly patients with no auditory complaints or risk for auditory deficiency. Stimuli were non-linear 80dB clicks. Sound stability was always above 80% and calibration was done daily.

It should be stated that tympanometry was done on the same day as TEOAE testing so that middle ear conditions would not yield altered recordings for reasons unrelated to the cochlea.29 The type A immitance curve was taken as normal according to Jerger’s classification.30 An Interacustic AZ26 digital middle ear analyzer calibrated according to ISO 8253/ IEC 645/ ISSO 389-1991 standards was used for this study.

### Data Analysis

PTA was based on the mean frequencies of 0.5, 1 and 2kHz. Thresholds up to 25dB were considered as normal; audiograms with thresholds over 30dB were considered as altered. Threshold lowering was evaluated for hearing loss over 3kHz; descending or notched curves were noted and maximum thresholds were recorded.

A response was positive in TEOAE testing when reproducibility was equal to or greater than 50% and the response amplitude was equal to or higher than 3dBNPS over noise in at least three consecutive frequencies.31

Response amplitudes were also compared as the signal-to-noise ratio (S/N) in dBNPS at 1 to 4kHz. Wilcoxon’s test was used for the comparative analysis of results by frequency between right and left ears. Mann-Whitney’s test was used to compare group test results. The significance level for the statistical tests was a = 0.05 (5%).

## RESULTS

Based on Wilcoxon’s test, a statistically significant difference was seen in the right and left ear auditory thresholds at 2kHz in the control group (p=0.027715). As a result of this finding, audiological results were analyzed separately for each ear.

Tables 1 and 2 show the descriptive statistical analysis (median, percentiles 25% and 75%, minimum and maximum) of the thresholds measures by PTA in groups GC1/GE1 and GC2/GE2. Also shown are the results of Mann-Whitney’s test, which was applied to compare pure tone thresholds between groups. Median values for each frequency and each ear are presented on Charts 1 and 2 for the control and study groups 1 and 2.

**Table 1:** Descriptive statistics for PTA results and Mann-Whitney’s test comparing audiometric thresholds in groups GC1 and GE1.

kHz	Ear	n		Median		25° Percentile		75° Percentile		Minimum		Maximum		p
		GC1	GE1	GC1	GE1	GC1	GE1	GC1	GE1	GC1	GE1	GC1	GE1	GC1 X GE1
0,25	RE	12	18	10	10	5	10	20	15	5	0	12,5	50	0,294648
	LE	12	18	10	10	0	10	20	15	10	5	15	35	0,637458
0,50	RE	12	18	10	10	0	5	15	20	7,5	0	10	25	0,110430
	LE	12	18	10	12,5	5	5	20	25	7,5	0	10	35	0,324659
1	RE	12	18	5	10	0	5	50	40	5	0	10	55	0,083769
	LE	12	18	10	17,5	5	10	15	35	5	0	15	65	0,385302
2	RE	12	18	7,5	27,5	0	10	15	45	2,5	0	10	85	0,149703
	LE	12	18	10	30	0	5	50	45	10	0	15	75	0,560150
3	RE	12	18	5	10	0	10	15	15	0	0	10	20	,027777*
	LE	12	18	10	10	0	5	20	15	0	0	10	35	,048406*
4	RE	12	18	15	10	0	5	25	20	5	0	15	25	0,275131
	LE	12	18	10	5	0	5	30	30	5	0	17,5	45	,037887*
6	RE	12	18	12,5	15	5	5	25	30	7,5	0	15	55	,040476*
	LE	12	18	15	17,5	0	15	25	35	7,5	0	25	60	0,099114
8	RE	12	18	10	22,5	0	15	35	55	5	5	17,5	95	0,163682
	LE	12	18	10	22,5	0	10	40	50	7,5	0	25	75	0,146332

**Key: dB** = decibel, kHz = kilohertz, RE = right ear, LE = left ear, n = number of valid cases, GC1 - control group 1, GE1 - study group 1, p = probability

* p < 0.05 - statistically significant

**Table 2:** Descriptive statistics for PTA results and Mann-Whitney’s test comparing audiometric thresholds in GC2 and GE2.

kHz	Ear	n		Median		25° Percentile		75° Percentile		Minimum		Maximum		p
		GC2	GE2	GC2	GE2	GC2	GE2	GC2	GE2	GC2	GE2	GC2	GE2	GC2 X GE2
0,25	RE	23	22	10	10	0	5	25	15	0	0	15	20	0,708336
	LE	23	22	10	10	0	5	20	15	10	0	15	20	0,990518
0,50	RE	23	22	5	10	0	5	20	15	5	0	10	30	0,029870*
	LE	23	22	10	10	0	5	20	20	5	0	15	30	0,342128
1	RE	23	22	5	10	-5	5	25	25	0	0	5	65	0,066453
	LE	23	22	5	12,5	0	5	45	30	5	0	15	65	0,340890
2	RE	23	22	5	20	0	10	25	35	0	0	10	65	0,055619
	LE	23	22	10	10	0	0	40	35	0	0	15	75	0,548243
3	RE	23	22	5	10	0	10	50	15	5	0	15	25	0,562266
	LE	23	22	15	10	0	10	60	15	5	0	20	25	0,954192
4	RE	23	22	10	10	0	5	65	15	5	0	25	45	0,654176
	LE	23	22	15	10	0	5	60	15	10	0	30	60	0,621907
6	RE	23	22	20	10	0	5	65	20	10	0	35	60	0,890691
	LE	23	22	20	10	0	10	60	25	15	0	35	60	0,828157
8	RE	23	22	15	22,5	0	10	70	40	0	0	25	60	0,953930
	LE	23	22	15	12,5	0	5	65	30	5	-5	30	65	0,927180

**Key: dB =** decibel, kHz = kilohertz, RE = right ear, LE = left ear, n = number of valid cases, GC2 - control group 2, GE2 - study group 2, p = probability

* p < 0.05 - statistically significant

The comparison of PTA thresholds between the control and study groups in which there was no exposure to noise, based on Mann-Whitney’s test (GC1 and GE1), revealed statistically significant differences at 3kHz (p=0.027777) and 6kHz (p=0.040476) in the right ear, and 3kHz (p=0.048406) and 4kHz (p=0.037887) in the left ear. The comparison between groups in which there was exposure to noise (GC2 and GE2) revealed a statistically significant difference only at 0.5kHz (p=0.029870) in the right ear. The analysis of PTA thresholds in all four groups made it possible to classify the audiograms and to characterize auditory changes in this sample. Table 3 shows these results.

**Figure 1:**
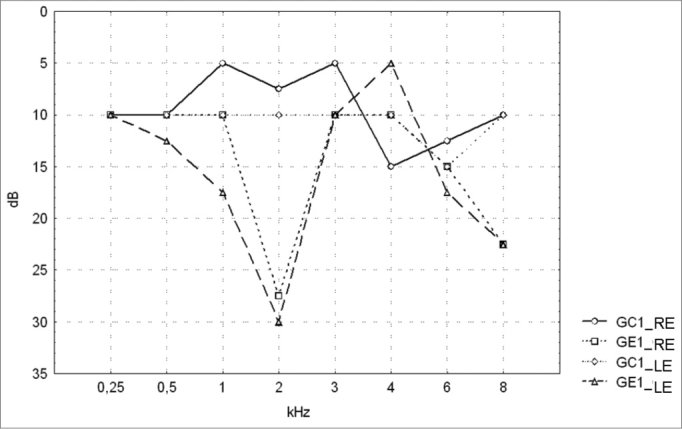
Pure tone threshold median values between 0.25 and 8kHz in the right and left ears for groups GC1 and GE1. - GC1_RE - control group 1 right ear; GE1_RE - study group 1 right ear; GC1_LE - control group 1 left ear; GE1_LE - study group 1 left ear

**Figure 2:**
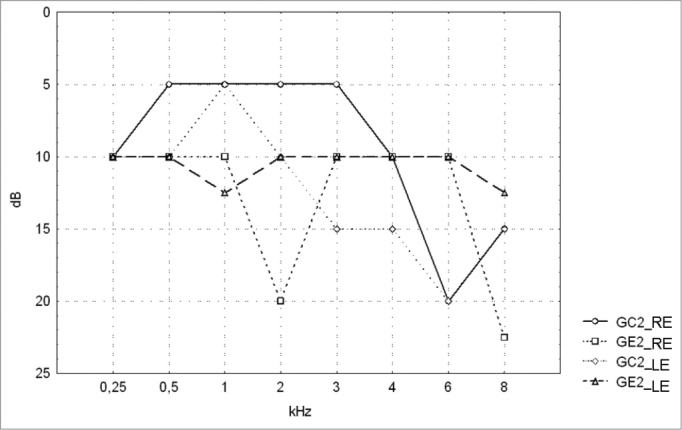
Pure tone threshold median values between 0.25 and 8kHz in the right and left ears for groups GC2 and GE2. - GC2_RE - control group 2 right ear; GE2_RE - study group 2 right ear; GC2_LE - control group 2 left ear; GE2_LE - study group 2 left ear

**Figure 3:**
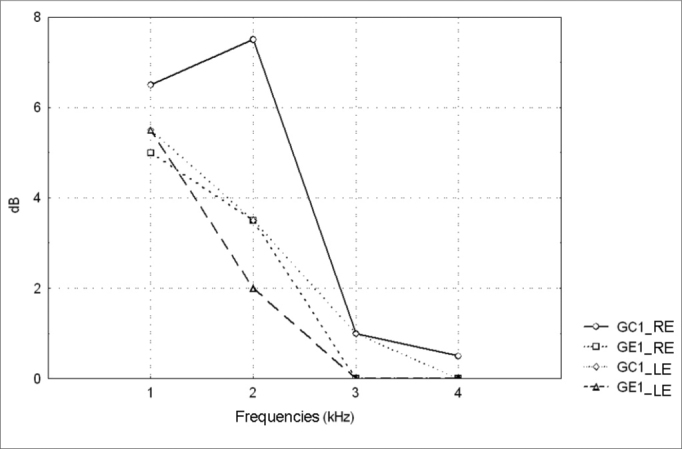
TEOAE amplitude median values (dB) between 1 and 4kHz in the right and left ears for groups GC1 and GE1 - GC1_RE - control group 1 right ear; GE1_RE - study group 1 right ear; GC1_LE - control group 1 left ear; GE1_LE - study group 1 left ear

**Figure 4:**
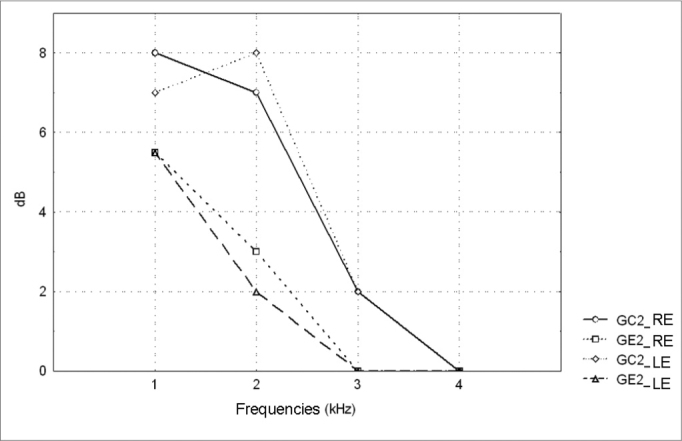
TEOAE amplitude median values (dB) between 1 and 4kHz in the right and left ears for groups GC2 and GE2. - GC2_RE - control group 2 right ear; GE2_RE - study group 2 right ear; GC2_LE - control group 2 left ear; GE2_LE - study group 2 left ear

**Table 3:** Analysis of PTA results for groups GC1, GC2, GE1 and GE2 considering the presence of hearing loss and the frequency that was affected.

Audiogram											
						Altered					
Groups	N	Normal		Mean only		Only at 3kHz and over		Mean + over 3kHz		Total	
		n	%	n	%	n	%	n	%	n	%
GC1	12	10	83	-	-	2	17	-	-	2	17
GE1	18	4	22	1	5,5	12	67	1	5,5	14	78
GC2	23	12	52,1	1	4,4	9	39,1	1	4,4	11	47,9
GE2	22	10	45,5	1	4,5	10	45,5	1	4,5	12	54,5

**Key: GC1-** control group 1; GC2 - control group 2; GE1- study group 1; GE2 - study group 2; n- number of cases

Tables 4 and 5 show the descriptive statistical analysis (median, percentiles 25% and 75%, minimum and maximum values) for right and left ear TEOAE responses at 1 and 4kHz for groups GC1/GE1 and GC2/GE2 and the results of Mann-Whitney’s test. This test aimed to compare TEOAE responses between groups. Charts 3 and 4 show median values at each tested frequency and ear in control and study groups 1 and 2.

**Table 4:** Descriptive statistics for TEOAE amplitudes and the results of Mann-Whitney’s test for comparing group GC1 and GE1 amplitudes.

kHz	Ear	n		Median		25° Percentile		75° Percentile		Minimum		Maximum		p
		GC1	GE1	GC1	GE1	GC1	GE1	GC1	GE1	GC1	GE1	GC1	GE1	GC1 X GE1
1	RE	12	18	6.5	5	1.5	0	11.5	10	-1	-5	26	17	0,384697
	LE	12	18	5.5	5.5	1.5	0	13	9	-5	-4	20	17	0,406840
2	RE	12	18	7.5	3.5	2	0	11.5	9	-2	-3	15	15	0,297235
	LE	12	18	3.5	2	0	0	12.5	6	-1	-3	15	13	0,312446
3	RE	12	18	1	0	0	0	8.5	6	-5	-5	11	11	0,554087
	LE	12	18	1	0	-0.5	0	5	4	-3	-4	13	16	0,619436
4	RE	12	18	0.5	0	0	0	5.5	4	-3	-4	10	10	0,347854
	LE	12	18	0	0	0	0	2	4	-5	-5	9	10	0,911988

**Key: dB =** decibel, RE = right ear, LE = left ear, n = number of valid cases; GC1 - control group 1; GE1 - study group 1, p = probability

p < 0.05 - statistically significant

**Table 5:** Descriptive statistics for TEOAE amplitudes and the results of Mann-Whitney’s test for comparing group GC2 and GE2 amplitudes.

kHz	Ear	n		Median		25° Percentile		75° Percentile		Minimum		Maximum		p
		GC2	GE2	GC2	GE2	GC2	GE2	GC2	GE2	GC2	GE2	GC2	GE2	GC2 X GE2
1	RE	23	22	8	5.5	4	0	14	9	-3	0	22	21	0,074991
	LE	23	22	7	5.5	5	0	14	12	-4	-5	20	17	0,190487
2	RE	23	22	7	3	3	-1	11	8	-3	-5	15	13	0,098823
	LE	23	22	8	2	6	0	11	6	-4	-5	16	12	0,004565*
3	RE	23	22	2	0	0	-1	5	4	-3	-5	17	12	0,074051
	LE	23	22	2	0	0	0	6	4	-2	-1	13	10	0,235185
4	RE	23	22	0	0	0	0	4	3	-5	-5	13	12	0,687187
	LE	23	22	0	0	0	-1	3	1	-5	-5	11	6	0,692119

**Key: dB =** decibel, RE = right ear, LE = left ear, n = number of valid cases; GC2 - control group 2; GE2 - study group 2, p = probability

* p < 0.05 - statistically significant

The Mann-Whitney test revealed that TEOAE responses in groups GC1 and GE1 were not statistically significant different from each other. There was a statistically significant difference in groups GC2 and GE2 in the left ear at 2KHz (p=0.004565).

Table 6 presents the comparative analysis of present or absent TEOAE responses in groups GC1, GC2, GE1 E GE2.

**Table 6:** Comparative analysis of TEOAE results in groups GC1, GC2, GE1 and GE2 for the presence or absence of responses.

Results of TEOAE			Groups		
	GC1	GE1		GC2	GE2
Bilateral absence	58,3%	66,6%		47,8%	72,7%
Unilateral absence	8,3%	11,1%		17,3%	9%
Bilateral presence	33,3%	22,2%		34,7%	18,1%

**Key: TEOAE -** transient evoked otoacoustic emissions, GC1 - control group 1, GC2 - control group 2, GE1 - study group 1, GE2 - study group 2

The Mann-Whitney test revealed that there was no significant difference in PTA thresholds in groups GE1 and GE2 (Table 7). A similar Mann-Whitney test analysis was done to compare TEOAE responses in study groups GE1 and GE2; no significant difference was found (Table 8).

**Table 7:** Results Mann-Whitney’s statistical test in comparing audiometric thresholds at 0.25 to 8kHz in groups GE1 and GE2.

GE1 x GE2				
kHz	RE		LE	
	Z	p	Z	p
0,25	0,54714	0,584286	-0,41304	0,679583
O,50	1,269294	0,204345	0,056541	0,954911
1	1,138661	0,254853	0,887149	0,375005
2	0,247955	0,804170	0,222965	0,823564
3	0,925221	0,354858	0,796668	0,425650
4	0,617838	0,536686	1,209549	0,226461
6	0,88853	0,374262	0,806474	0,419976
8	1,098379	0,272047	1,038404	0,299090

**Key:** RE - right ear, LE - left ear, Z = test statistics, p = probability

**Table 8:** Results Mann-Whitney’s statistical test in comparing TEOAE amplitudes in groups GE1 and GE2.

GE1 x GE2				
kHz	RE		LE	
	Z	p	Z	p
1	-0,50542	0,613268	-0,50542	0,613268
2	0,600612	0,548102	0,600612	0,548102
3	0,806896	0,419732	0,806896	0,419732
4	0,360074	0,718794	0,360074	0,718794

**Key:** RE - right ear, LE - left ear, Z = test statistics, p = probability

The correlation coefficients between duration of alcohol consumption and pure tone thresholds and TEOAE recordings for groups GE1 and GE2 are shown on Tables 9 and 10.

**Table 9:** Correlation coefficients for pure tone thresholds and the duration of alcohol use in groups GE1 and GE2, for each frequency range that was investigated.

PURE TONE FREQUENCIES (Hz)		GE1 + GE2	GE1	GE2
RE	250	-0,08	-0,06	-0,06
	500	-0,09	-0,00	-0,15
	1000	-0,06	0,10	-0,16
	2000	-0,20	0,11	-0,52*
	3000	-0,23	0,20	-0,58*
	4000	-0,16	0,18	-0,45*
	6000	-0,00	0,33	-0,30
	8000	0,01	0,32	-0,23
LE	250	-0,15	-0,17	-0,16
	500	0,12	0,41	-0,16
	1000	0,01	0,33	-0,20
	2000	-0,13	0,22	-0,45*
	3000	-0,11	0,24	-0,45*
	4000	-0,06	0,16	-0,22
	6000	-0,07	0,08	-0,22
	8000	-0,01	0,17	-0,16

**Key:** RE - right ear, LE - left ear, GE1 - study group 1, GE2 - study group 2

* p < 0.05 - statistically significant

**Table 10:** Correlation coefficients for TEOAE amplitudes and the duration of alcohol use in groups GE1 and GE2, for each frequency range that was investigated.

Frequency range (Hz)		GE1 + GE2	GE1	GE2
RE	1000	-0,07	-0,15	-0,03
	2000	-0,15	-0,28	-0,00
	3000	-0,06	-0,35	0,26
	4000	-0,17	-0,18	-0,14
LE	1000	0,06	-0,19	0,02
	2000	-0,06	-0,18	0,08
	3000	-0,04	-0,26	0,03
	4000	-0,11	-0,19	-0,03

**Key:** TEOAE - transient evoked otoacoustic emissions, GE1 - study group 1, GE2 - study group 2, RE - right ear, LE left ear

## DISCUSSION

Studies in the literature that have investigated alcohol ototoxicity have not shown clearly the toxic potential of alcohol on the auditory system or which might be the probable site of injury, if applicable. Itoh et al.32 have shown that continuous consumption of alcohol does not increase auditory risk. Nakamura et al.,^33^ on the other hand, found a significant relation between the use of alcohol and idiopathic hearing loss. Popelka et al.^34^ demonstrated that subjects with a history of daily consumption of alcohol are at a higher risk for high frequency hearing loss.

Research in this are is limited, not only because hearing loss in alcoholics is a minor problem for such individuals, but also due to the difficulty of excluding other interfering factors - such as noise - that increase the risk of hearing loss, as reported by Nordhal.^25^

A comparison of PTA thresholds between groups based on Mann-Whitney’s test using median, minimum and maximum values (Table 1 and Chart 1) revealed that alcoholic subjects (GE1) had statistically significantly higher auditory thresholds at high frequencies compared to the control group (GC1). On the other hand, in those groups in which occupational noise exposure was a variable under analysis, descriptive statistics (Table 2 and Chart 2) was inconclusive about whether auditory thresholds in alcoholic subjects (GE2) are higher than auditory thresholds in non-alcoholic subjects exposed to noise (GC2). A comparison between the results obtained in alcoholic subjects with no occupational noise exposure and the control group (GC1 and GE1) suggests that the toxic effect of alcohol occurs in the basal portion of the cochlea, where maximum vibration of the basilar membrane due to high frequency sounds takes place. These groups were matched for age, where use of alcohol was the only probable risk factor for hearing. The abovementioned finding, however, is unclear in the noise-exposed groups (GC2 and GE2); here, the harmful effect of noise on hearing was found in both groups, which reduced the differences between groups GC2 and GE2 compared to groups GC1 and GE1, where only alcohol consumption was compared.

PTA may characterize the ototoxic effect of alcohol in each subject for each group. There was a higher rate of hearing loss in alcoholic subjects in groups with no exposure to noise compared to the control group, for a normal threshold value below or equal to 25dBNA (Table 3). These results support previous findings stating that middle and high frequencies - particularly high frequencies (over 3kHz) - are more affected in alcoholic individuals. The occurrence of hearing loss was similar in the noise-exposed (Table 3). The difference allows no conclusive analysis about alcohol toxicity on the auditory system of these subjects.

Our findings in subjects where the single risk factor for hearing was alcohol abuse confirm the initial assumption that suggests a relation between alcoholism and hearing loss.

A comparison between the current study and others shows that, except for Nordahl,^25^ who attributed hearing loss to noise exposure, all the other studies described high frequency hearing loss in alcohol abusers. These studies, however, did not control for variables such as age and exposure to other toxic substances, as was done in the current study.^1^, ^3^, ^5^, ^6^, ^7^, ^8^, ^9^, ^10^, ^11^, ^12^, ^13^, ^14^, ^15^, ^16^, ^17^, ^18^, ^19^, ^20^, ^21^, ^22^

A comparison of TEOAE responses between groups revealed a statistically significant difference at 2kHz in the left ear in subjects exposed to noise; the TEOAE signal-to-noise ratio was lower in alcoholic subjects (Table 5). There are, however, no data in the current study to justify this finding. We found no published papers that had analyzed TEOAE recordings in control and alcoholic groups that could be compared with the current study.

TEOAE results for each subject in all the groups reinforce PTA thresholds, which is not the case of mean response times. In other words, all of the groups confirm PTA observations that alcohol is ototoxic.

There was a higher rate of absence of unilateral and bilateral TEOAE in alcoholic subjects not exposed to noise, similar to alcoholic subjects exposed to noise (Table 6). It should be noted that absence of TEOAE in GC1 may be due to aging, given that control group subjects were aged between 40 and 57 years.

TEOAE are absent more in alcoholic subjects with or without noise exposure compared to non-alcoholic subjects. This finding is still clearer upon analysis of TEOAE recordings in alcoholic subjects with no exposure to noise (GE1) where alcohol the only auditory risk factor, given that groups were paired for age. There is one published paper by Niedzielska et al.5 in which TEOAE were also used in the evaluation; the result are similar to those in the current study.

Another important finding is the site of injury. Although studies on alcohol abusers have found high frequency hearing loss, questions remain about the auditory structure that might be affected. In most of these studies PTA was used for evaluation. Our findings suggest that the cochlea is affected in alcoholic subjects, as otoacoustic emissions are evidence of functional external hair cells in the organ of Corti. This assumption is in agreement with Ylikoski’s^14^ description, where studies on the temporal bones and the brainstem of alcoholic subjects have shown that loss of cochlear neurons are usually accompanied by advanced loss of hair cells.

The analysis of PTA and TEOAE results to compare groups GE1 and GE2, based on Mann-Whitney’s test (Tables 7 and 8), did not reveal significant differences. This analysis aimed to find whether simultaneous exposure to both harmful factors would potentialize the effect of each other on the auditory system. The findings revealed that this hypothesis could not be confirmed.

Table 9 shows the correlation coefficient analysis between the variables use of alcohol and audiometric threshold results. The correlation was relatively low when calculated for the groups with and without associated noise. These correlations increase, however, when calculated separately in both groups. In this case a significant result was found for the right and left ears in group GE2 only for subjects also exposed to noise. This finding suggests that in our series, duration of alcohol abuse increased the probability of high frequency hearing loss. Our findings are similar to those of Wheeler et al.^12^, Golabek and Niedzielska, 18 and are different from those of Rossi;22 in the latter study, no correlation between duration of alcohol abuse and hearing loss was found on PTA.

A similar analysis focusing the amplitude of TEOAE responses (Table 10) revealed low correlations; when negative, these correlations indicated inverse relations between duration of alcohol abuse and TEOAE results. Thus, an increased duration of alcohol abuse resulted in decreased amplitude, although the correlations were not statistically significant. The most significant correlation was found in the study group with no noise exposure (Table 8). We found no published papers about evoked otoacoustic emissions in alcoholic subjects that analyzed the duration of alcohol abuse and hearing loss.

In general, the results of PTA and TEOAE testing revealed a possible relation between alcoholism and cochlear sensorineural hearing loss in alcoholic subjects.

## CONCLUSION

Based on our results in the present series, we concluded that there is a probable relation between alcoholism and cochlear sensorineural hearing loss at high frequencies. The association between exposure to alcohol and noise did not potentiate the effect of each other on the auditory system. Ototoxicity resulting from long-term exposure to alcohol abuse may affect cochlear function, harming specifically the external hair cells.
